# Threat of wheat blast to South Asia’s food security: An *ex-ante* analysis

**DOI:** 10.1371/journal.pone.0197555

**Published:** 2018-05-21

**Authors:** Khondoker Abdul Mottaleb, Pawan Kumar Singh, Kai Sonder, Gideon Kruseman, Thakur Prasad Tiwari, Naresh C. D. Barma, Paritosh Kumar Malaker, Hans-Joachim Braun, Olaf Erenstein

**Affiliations:** 1 Socioeconomics Program, CIMMYT (International Maize and Wheat Improvement Center), Texcoco, Mexico; 2 Global Wheat Program, CIMMYT, Texcoco, Mexico; 3 Geographical Information System Unit, CIMMYT, Texcoco, Mexico; 4 Country representative, CIMMYT, Dhaka, Bangladesh; 5 Wheat Research Centre, BARI, Bangladesh; Murdoch University, AUSTRALIA

## Abstract

New biotic stresses have emerged around the globe over the last decades threatening food safety and security. In 2016, scientists confirmed the presence of the devastating wheat-blast disease in Bangladesh, South Asia–its first occurrence outside South America. Severely blast-affected wheat fields had their grain yield wiped out. This poses a severe threat to food security in a densely-populated region with millions of poor inhabitants where wheat is a major staple crop and per capita wheat consumption has been increasing. As an *ex ante* impact assessment, this study examined potential wheat-blast scenarios in Bangladesh, India, and Pakistan. Based on the agro-climatic conditions in the epicenter, where the disease was first identified in Bangladesh in 2016, this study identified the correspondingly vulnerable areas in India, Pakistan and Bangladesh amounting to 7 million ha. Assuming a conservative scenario of 5–10% for blast-induced wheat production loss, this study estimated the annual potential wheat loss across the sampled countries to be 0.89–1.77 million tons, equivalent to USD 132–264 million. Such losses further threaten an already-precarious national food security, putting pressure on wheat imports and wheat prices. The study is a call for action to tackle the real wheat-blast threat in South Asia.

## Introduction

**Fig 1:**
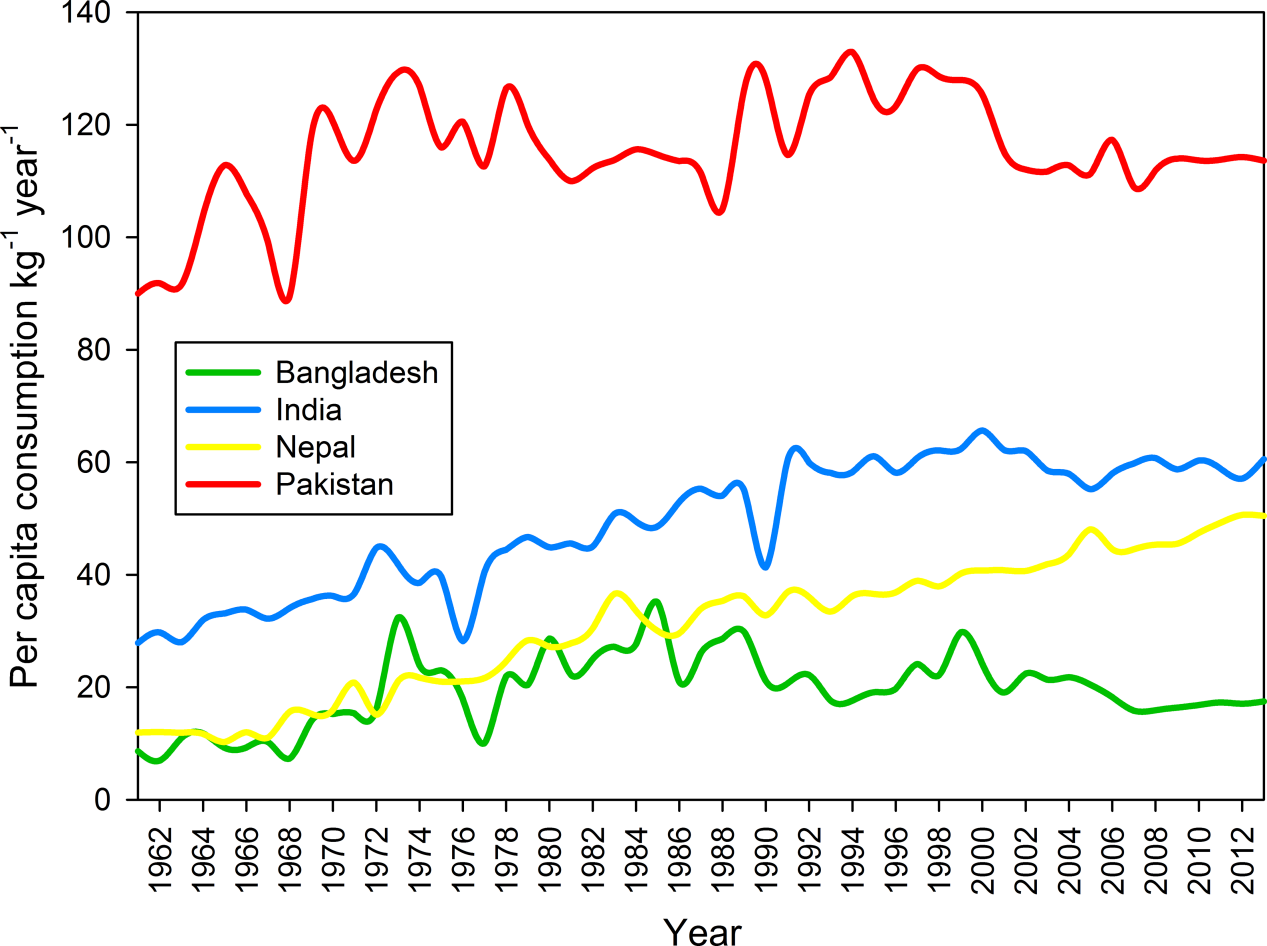
Wheat consumption trends (kg/capita/year) in selected countries in South Asia. Source: own calculations based on FAOSTAT [5].

Wheat consumption in South Asia’s traditional rice economies, such as Bangladesh, Nepal, and India, has been steadily increasing since the Green revolution in the 1960s now oscillating around 20 kg per capita per year in Bangladesh, 50–60 kg in Nepal and India; whereas, it has long been around 100 kg in Pakistan (Fig 1). This increasing preference for wheat is not unique to South Asia, as it has also become evident in the urban areas of sub-Saharan Africa in recent years [1, 2]. The global population is projected to increase 25–33% by 2050 from the current level [3] with more than 65% of the increase taking place in sub-Saharan Africa and South Asia [3]. These trends imply significant increases in food demands in general, including major staples, and wheat demand in particular [4].

The need to supply more wheat and other food grains to meet the increasing and changing demand in developing countries is increasingly challenging, given the rapidly-declining per capita arable land and renewable fresh water in these countries [6] with the added stresses of climate change. Additionally, new biotic stresses have emerged around the globe over the last decades, threatening food safety and production and overall food security in developing countries.

In 2016, the devastating wheat-blast disease caused by the fungus *Magnaporthe oryzae* pathotype *Triticum* (MoT) was reported in Bangladesh, South Asia [7–12]. This is a significant incident. First, it represents the first occurrence outside South America of this particularly aggressive disease. Second, it entered South Asia, where wheat is a major staple for the masses. In 2016, it was believed to be confined to Bangladesh, a densely-populated, land scarce country with nearly 160 million people, of which 18% are extremely poor, living on less than USD 1.90 day^-1^ [13]. In 2017, wheat-blast-like symptoms were unofficially reported for the first time from eastern India in various newspapers [14,15]. Although it has not been officially confirmed these disease symptoms were blast, the likelihood of wheat blast passing across the Bangladesh border is high. Bangladesh shares a border of 2,217 kilometers only with West Bengal, India, which cuts across the lower part of the Gangetic plains. This is an immense contiguous densely-populated and intensively cultivated area that stretches from Bangladesh to adjacent West Bengal up to north-western India and on to the Indus plains of Pakistan, home to the rice-wheat systems and the staple basket for much of South Asia [16–17].

Wheat blast was first reported in the Parana state of Brazil in 1985 [18]. Later it spread to the humid and warmer zones of Bolivia, Paraguay and north-eastern Argentina [19]. The disease is so destructive that it reduced wheat yield in Sao Paulo, Brazil in 2005 by 14–32%, even after two applications of fungicides [20]. In Bangladesh, in February 2016, wheat blast affected nearly 15,000 ha (3.5% of the total 0.43 million ha of wheat area in Bangladesh), with wheat yields in the affected fields reduced by 5–51% [9]. Any spread of wheat blast into the rest of Bangladesh and particularly into India and Pakistan, could wreak havoc on wheat production in South Asia and significantly threaten food security and the overall well-being of a region with millions of poor wheat consumers who depend on wheat as their main source of dietary energy.

The objective of this study was to examine wheat-blast scenarios in Bangladesh, India, and Pakistan by applying an *ex ante* impact assessment framework to quantify the potential impacts in South Asia’s wheat areas, which are potentially vulnerable to wheat-blast considering their favorable climate for the disease. This study intends to inform stakeholders of the wheat-blast threat in South Asia and assess the implications.

The study is organized as follows: the next section gives a brief perspective on the spread of wheat blast, its causes and implications; Section 3 includes the materials and methods section, including the selection of wheat blast hotspots, the current status of land allocation to wheat and wheat yield in the selected regions of the sampled countries; Section 4 presents simulation results and Section 5 concludes and provides recommendations.

## Wheat-blast disease

**Fig 2:**
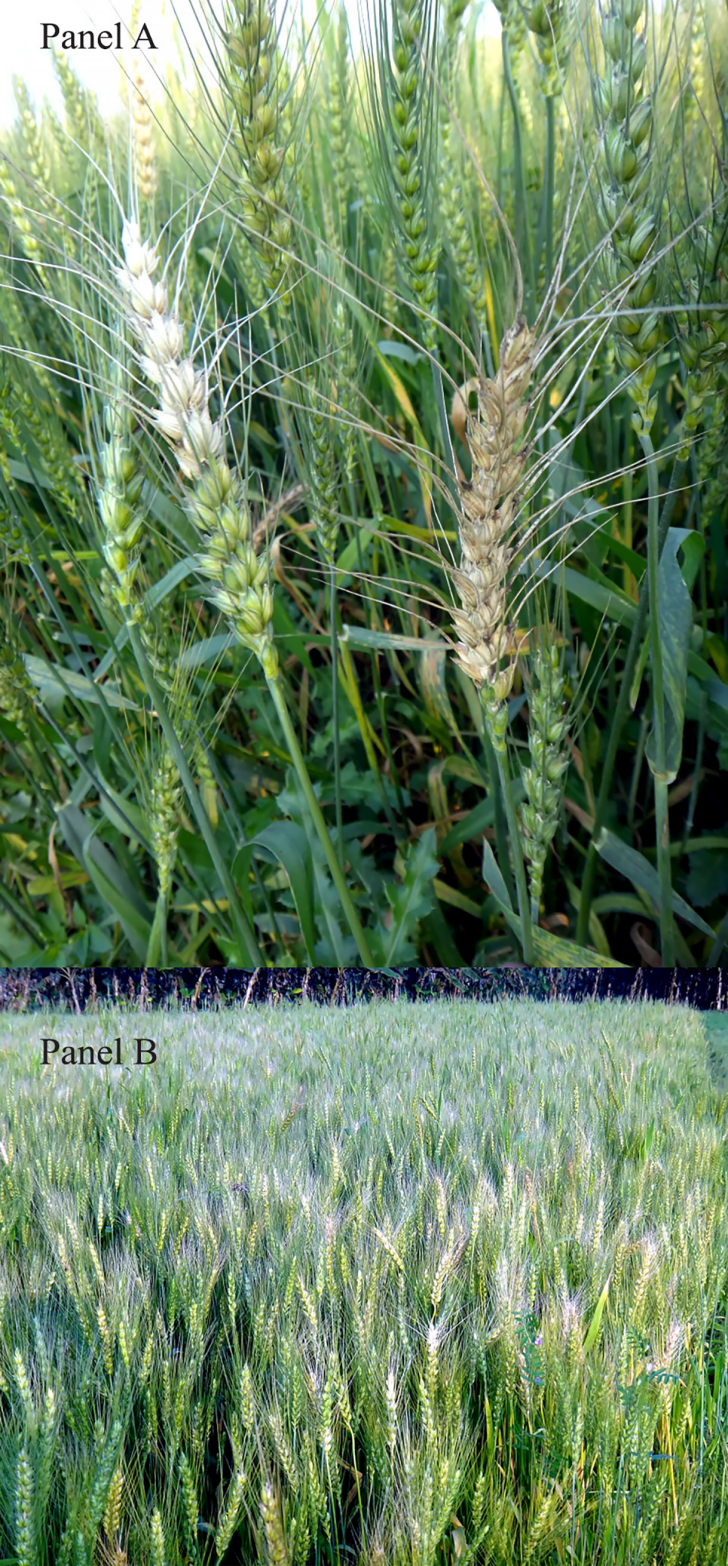
Wheat-blast symptoms in Jhenaidah district, Bangladesh in February 2017. **Panel A: Infected spikes; Panel B: Infected field.** Courtesy: Moksedul A. Arafat, Technical Officer, Jessore Hub, CIMMYT, Bangladesh, 2017.

Wheat blast is usually classified as a spike disease (Fig 2). It can be found on all the aerial plant parts of wheat [18], although leaf infection is seen only in highly-susceptible varieties [19, 21]. Spike infection is the most notable symptom of the disease [19], which is often confused with Fusarium head blight (FHB) infection; but rather than attacking individual spikelets and conferring a salmon or pinkish tint to flames as FHB usually does, blast attacks the rachis, leading to bleached spikelets above the point of infection and bright black spots on the rachis [19]. Grains from blast-infected heads are usually small, shriveled, deformed, and have low-test weight. The highest yield losses happen when head infections start during anthesis or early grain development stages [19].

The wheat-blast pathogen emerged only 32 years ago in South America, but has fully adapted to its new host ‒ wheat ‒ and continues to spread and evolve genetically. The first occurrence of this biotic stress outside South America represents a major event which, in turn, may trigger a new selection pressure, from the Bangladesh outbreak epicenter, on this highly-evolving fungus. Although the comparative genome analysis showed that isolates from diverse wheat regions in Bangladesh appeared clonal [7, 22], and pathogenomics analysis [9] confirmed that the Bangladeshi wheat-blast most likely arrived from South America, a change in pathogen population can be expected due to a new environment and hosts selection pressure. Bangladesh and South Asian agro-ecology, as well as the host wheat germplasm and weeds are quite different from that of South America. These differences will put different selection pressures on the pathogen and the fungus will likely evolve and cope with the new environment, which can trigger a new selection pressure in the pathogen population. Importantly, the strain identified in Bangladesh corresponds to a very aggressive type compared to *MoT* strains collected in the early days of observation when the disease was considered an oddity, unable to cause large and frequent epidemics [7, 23, 24]. In addition, wheat blast is a seed-borne disease [25] and could potentially spread due to seed sharing between farmers, as frequently observed in South Asia, and with wheat trade between countries. Moreover, it is also airborne [26] and can move relatively long distances during the cropping season. The 2016 detection of the wheat-blast in Bangladesh could thus spread further into India and Pakistan, not least as Bangladesh shares a long border with India. In 2017, unofficial media reported that spikes with wheat-blast-like symptoms had been observed in West Bengal, adjoining the western borders of Bangladesh [14–15].

At present, most of the commercially-grown wheat cultivars in South Asia are susceptible to wheat blast. Although some cultivars tolerant or moderately resistant to wheat blast have been identified in localized geographic areas, no durable resistant cultivar has been developed until today [19, 21]. The level of yield losses and speed of epidemics caused by *MoT* along with the lack of resistance may require innovative approaches to manage this disease. Generally, the known fungicides are ineffective under high disease pressure, and were only partially effective under moderate to low infection of the wheat-blast isolates in Brazil [24]. Moreover, the pathogen has shown a rapid ability to develop resistance to certain classes of fungicides [19, 23, 24]. Still, there remains a role for protectant low risk fungicides, also as the pathogen's evolution reflects the distinct selection pressure exerted by long term use of high risk fungicides (e.g. QoIs, DMIs or SDHIs as the sole molecule). Indeed, the wheat-blast isolate in Bangladesh was effectively controlled with Tebuconazole and Trifloxystrobin, which is widely available in South Asia [Malakar, personal communication]. Nevertheless, the list of approved and locally available fungicides in South Asia is limited and, therefore, testing the active-ingredient efficacy is required. At present isolated incidences of fields affected by wheat-blast-like symptoms are dealt with by burning the standing crop. This destructive method of dealing with the disease can lead to severe production shortfalls if the disease becomes more pervasive. Moreover, in addition to similar agro-climatic conditions across Bangladesh, India and Pakistan, the changing global climate and the evolving pathogen (e.g. increasing aggressiveness, fungicide resistance and sexual recombination) can further aggravate disease incidence with the likely expansion to other major wheat-producing countries.

Duveiller et al. [27] reported that there are wheat-producing regions in the world, including central India, Bangladesh and Ethiopia, where the disease had not yet been reported, but the agro-climatic conditions are similar to regions in South America where the disease occurs. The 2016 occurrence of wheat blast in Bangladesh fits the original prediction. Maciel [21] expressed a similar concern, pointing out that wheat blast may spread to European countries, Canada, and the USA under the scenarios of global warming due to climate change. Cruz et al. [28] predicted that in the USA wheat blast could break out in 25% of the wheat area, with a probability exceeding 70% in the warmer and more humid states. Hence a serious threat of further wheat-blast expansion exists.

Wheat production in Bolivia is a notorious example of how wheat blast can destroy a nation’s wheat crop. In the early 1990s, Bolivia started an initiative to increase its national wheat production, mainly to attain wheat food self-sufficiency [29]. The country managed to increase wheat production from 10,865 tons in 1989 to 75,435 tons in 1994, and 120,414 tons in 1997 [29]. However, the outbreaks of wheat blast in the subsequent years caused tremendous yield losses; consequently, the production volume dropped to 37,750 tons in 1999 from 172,892 ha [29]. Many resource-poor farmers were discouraged by the wheat-blast threat and the area under wheat fell in subsequent years [5].

## Materials and methods

**Fig 3:**
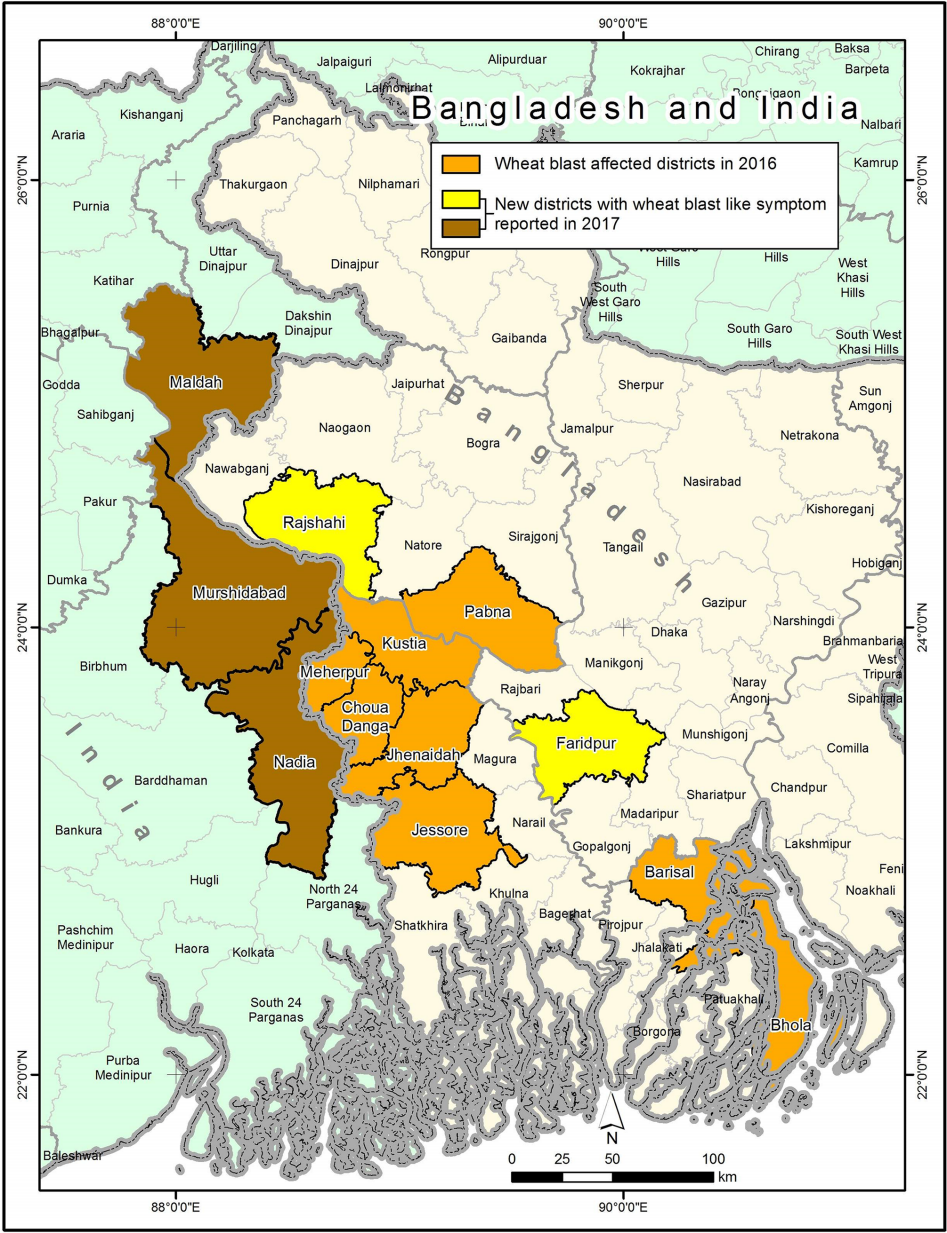
Original districts with wheat-blast in 2016 and new districts with wheat-blast like symptoms in 2017 wheat season in Bangladesh and eastern India. Source: Bangladesh 2016 [9]; 2017 (authors’ observations); India [14, 15].

We use a climate analogue model to first identify prospective wheat blast hotspots in South Asia. As evidenced in South America, warm and humid areas are the most vulnerable to wheat-blast incidence [8]. Bangladesh has a sub-tropical monsoon climate across its primarily low-elevation landscape, with wide seasonal variations in rainfall, high temperatures and humidity. Wheat is grown during the relatively cool and dry winter. In March 2016, the emergence of wheat blast disease was first officially-recognized in Bangladesh, that reportedly affected eight districts (Fig 1): Chuadanga, Meherpur, Jessore, Jhenaidah, Bhola, Kushtia, Barisal and Pabna [9]. A team of Food and Agriculture Organization of the United Nations (FAO), however visited 39 spots in seven districts and assessed the disease severity [30]. The wheat-blast affected sites in Bangladesh were identified using geo-referenced information from the FAO-led field study [30]. These sites were used to generate an analogue climate map applying the analogue tool. Using long term temperature and rainfall patterns (1960–1990) in the most severely affected geo-spatial coordinates of the plots in four districts reported: Bhola, Chuadanga, Jhenaidah and Meherpur [30], the present study characterizes the wheat-blast-vulnerable agro-climate, and identifies the homologue areas (hotspots) in Bangladesh, India and Pakistan by matching these characteristics during the wheat growing season (Fig 3).

**Fig 4:**
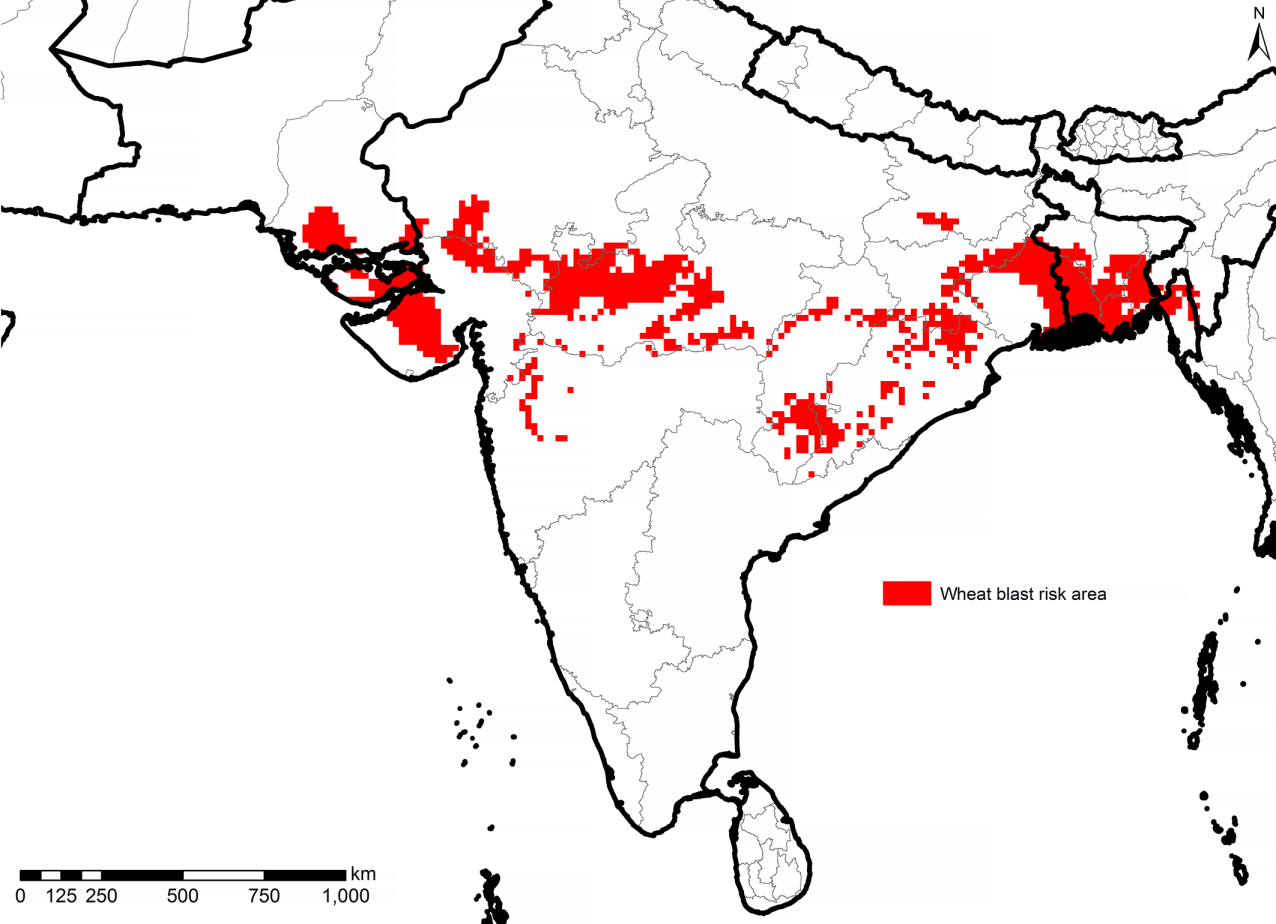
South Asia wheat-blast vulnerability map. Source: Authors’ own estimation.

The analogue climate approach has been utilized for a number of studies mainly focused on climate change analysis [31–37]. The analogue tool used here is based on R code. It analyzes climate variables at a given location and takes into account rainfall and average temperatures as variables (in combination or each factor by itself) and uses spatial analysis to identify places in the target areas that have a similar (analogue) climate based on a weighted similarity index. Both variables can be weighted depending on the specific nature of the analysis needed (e.g. disease risk, crop aptitude, biotic and abiotic stress-adapted landraces, representativeness of trial sites, etc.). This analysis can be performed with the current climate data to identify current spatial climate analogues as well as with predicted future climate data, be it looking forward or backward to identify either the future climate of a location or where future predicted climate can be found elsewhere [38]. For the present study, we identified spatial analogue areas for January and February’s climate having the highest importance for the emergence of the wheat-blast disease for the Bangladesh outbreak. The spatial scope of the analysis was limited to India, Pakistan and Bangladesh. The gridded outputs for all locations with similarity indices above 0.6 (60%) were merged in a GIS and used to identify wheat-producing districts at risk of wheat-blast infection (Fig 4).

The initial 2016 event in Bangladesh affected 5.4% of its potentially-vulnerable areas with an average yield loss of 24.5% in the affected fields, representing an aggregate production shock of 1.3% for the vulnerable areas. The 2016 event implied a particularly favorable scenario for wheat blast in terms of high humidity and temperature at the right time of the wheat growth stage (prior and during heading) with the initial presence of the blast inoculum. One may assume that now having the disease endemic in South Asia increases the prospects of it emerging again elsewhere in the region when the conditions are right. It also is likely that wheat blast will persist in the presence of alternative hosts (e.g. wild grass species, non-wheat crops), which undermine the prospects of eradicating the disease.

The present study uses a simple *ex ante* analysis to assess wheat-blast scenarios in South Asia. We consider two scenarios applied across the prospective wheat blast vulnerable areas: a 5% and a 10% wheat-blast-induced wheat-production shock in the vulnerable areas only; with in each case no effect (0% loss) in the non-vulnerable areas. One can interpret the 5% scenario as an average scenario; for instance, 10% of the vulnerable area could be affected by a 50% yield loss; alternatively, 5% of the vulnerable area could be affected by a devastating 100% yield loss, or variations thereof. Lacking further detailed insights for now, we assume the 5% and 10% production shocks to be reasonable scenarios within an *ex ante* impact assessment framework.

An additional but important caveat is that these are potential wheat-blast-induced production losses in the vulnerable areas during affected years. These scenarios depict specific situations where the season’s agro-climatology favors an outbreak of blast in the vulnerable areas (i.e. the simultaneous combination of high humidity and temperature at the right time in the wheat season and in the presence of blast inoculum). Such conducive instances for wheat blast do not occur in all years, even in the vulnerable areas. The scenarios thereby represent occurrences that may happen in one particular year simultaneously across South Asia–but not necessarily every year in the vulnerable areas. Even if it occurs, the disease may present itself as is the case in the 2016–17 wheat season in Bangladesh, which was less propitious for wheat blast and hence was less badly affected than in the preceding 2015–16 season. However, even though losses in the 2016–17 cycle were less than in 2015–16, the occurrence of the disease in Bangladesh indicates that wheat blast is now established, and occurs even though the weather was not conducive (relatively dry, limited rain).

## Results

In Bangladesh, matching with the climate in the original four severely wheat-blast affected districts, the present study identifies 0.28 million ha (out of a total 0.43 million ha under wheat production) [39], as vulnerable to wheat blast located in 46 districts (out of total 64 districts—Table 1). Applying the same historical weather-variable matching technique, this study shows that, in India, out of 30.96 million ha of actual wheat area [40], 6.57 million ha are found to be vulnerable to wheat-blast disease located in 138 districts in 11 states. In contrast, out of 9.52 million ha of the total wheat area in Pakistan, only five districts located in Sindh Province with an approximate area 0.14 million ha [41], are vulnerable to wheat-blast disease.

**Table 1:** Wheat-blast vulnerability indicators in Bangladesh, Pakistan and India.

Country	Current wheat area (million ha)	Vulnerable states /divisions	Vulnerable districts (#)	Vulnerable area (million ha)	Current wheat production (million ton)	Potential wheat loss (‘000 ton) (million USD) under different wheat-blast induced production shocks (%)	
						5%	10%
Bangladesh	0.43^a^	Divisions: Barisal, Chittagong, Dhaka, Khulna, Sylhet and Rajshahi	46	0.28(65%)	1.25 ^a^	42.6 (USD 6.3)	85.1(USD 12.7)
India	30.96 ^b^	States: Andhra Pradesh, Bihar, Chhattisgarh, Gujarat, Jharkhand, Madhya Pradesh, Maharashtra, Mizoram, Odisha, Rajasthan, Tripura, and West Bengal	138	6.57(21%)	95.85 ^b^	820.5(USD 122.3)	1,640.9(USD 244.5)
Pakistan	9.52^c^	State: Sindh	5	0.14(1.59%)	26.96^c^	22.7(USD 3.4)	45.3(USD 6.8)
Aggregate South Asia	40.85		195	6.99(17.1%)	124.1	886(USD 132)	1,771(USD 264)

Authors’ calculation.

^a^BBS [39].

^b^Government of India [40].

^c^ Pakistan Agriculture Information System [41].

Note: Price of wheat set at USD 149/ton

The vulnerable area, therefore, stretches in a broad band across the sub-tropics of the Indian sub-continent: from the southern half of Bangladesh into India’s West Bengal and on to India’s Gujarat in the West and into Pakistan’s southern Sindh (Fig 4). The vulnerable area represents a vast wheat-producing area totaling 7 million ha. Still, most of the wheat-producing areas across the Indo-Gangetic plains remain relatively spared due to their more northern location and correspondingly cooler winter season.

Table 1 also presents the wheat-blast vulnerability indicators for Bangladesh, India and Pakistan, including current wheat indicators, predicted vulnerability indicators and the potential wheat loss from wheat blast for two scenarios (assuming 5% and 10% wheat-blast-induced wheat-production shocks in the vulnerable areas). Table 1 shows that Bangladesh is the most vulnerable to wheat blast in South Asia: 65% of the total wheat area is vulnerable, reflecting the sub-tropical climate. Still, Bangladesh is the much smaller wheat producer among the three South Asian countries in terms of area and production. This would potentially result in an estimated wheat loss of 43–85 thousand tons in affected years under the 5–10% scenarios, equivalent to USD 6.3–12.7 million when valuing wheat at USD 149/ton.

Compared to Bangladesh’s 65%, only 21% of India’s total wheat area is vulnerable to wheat blast (Table 1), reflecting that wheat is more widely grown in the colder northern latitudes. Still, the sheer size of India’s wheat area and production implies, that even a fifth being vulnerable has potentially dire consequences, were wheat blast to spread across India. This would result in an estimated potential wheat loss of a whopping 0.8–1.6 million tons in the affected years under the 5–10% yield loss scenarios, equivalent to USD 122–244 million (again valuing wheat at USD 149/ton).

Table 1 shows that Pakistan is the least vulnerable to wheat blast in South Asia: 1.6% of the total wheat area could be vulnerable, reflecting the more northern latitude of its wheat production with the vulnerable area confined to Sindh in the south. Therefore, although wheat is the major staple in Pakistan and its wheat area and production is second to India in South Asia, potential blast-induced losses are the lowest for Pakistan. Wheat blast could potentially result in an estimated wheat loss of 23–45 thousand tons in affected years under the 5% and 10% scenarios, equivalent to USD 3.4–6.8 million.

Across South Asia, the wheat-blast-induced wheat-yield loss scenarios of 5–10% imply a potential wheat loss of 0.89–1.77 million tons in affected years, equivalent to USD 132–264 million. The threat of wheat blast increases the pressure on the already-precarious national food security in South Asia, and potentially adds pressure on wheat imports and wheat prices. Although wheat is one of the major and increasing sources of dietary calories in Pakistan, India and Bangladesh. Pakistan and Bangladesh are already regular importers of wheat over the last decade and, in 2016, even India resorted to imports. A potential wheat-blast-induced reduction of wheat production would have a significantly negative impact on the overall food security in the region and on international trade, potentially feeding into international wheat-price increases and threatening the food security of the poorest wheat consumers.

The yearly per capita consumption of wheat in Bangladesh, India and Pakistan in 2013 was 17.5 kg, 61 kg and 114 kg respectively (Table 2). Bangladesh, to meet wheat demand, imported 2.84 million tons [5] of wheat, on average, from 2009 to 2013. Under the potential wheat-blast-induced production losses of 5% and 10%, to maintain the consumption at the 2013 level, Bangladesh needs to import 1.5–3.0% more wheat. Pakistan, a net importer of wheat based on the net average wheat trade statistics, imported 0.22 million tons of wheat [5] from 2009 to 2013. To maintain wheat consumption at the 2013 level in Pakistan, under the potential wheat-blast-induced production losses of 5–10%, the country needs to increase wheat imports by 10–21%. In contrast, based on the average of net wheat trade from 2009 to 2013, India was a net exporting country of wheat, exporting a yearly average of 2.24 million tons of wheat. Under India’s 2009–13 wheat trade balance, the potential wheat-blast-induced production losses of 5% and 10% would reduce wheat exports by 37–73%. In 2016 India resorted to importing wheat, but normal circumstances would keep India as net exporter, although wheat-blast-induced losses could jeopardize India’s reserve stocks.

**Table 2:** Implications of wheat-blast scenarios for net wheat trade in Bangladesh, India and Pakistan.

Country	Yearly actual per capita/kg consumption of wheat (year 2013)	Net wheat trade 2009–13 average (million tons, import (-), export (+)	Potential implications for wheat trade (relative change [relative increase (+), decrease (-)] of 2009–13 average balance) under different wheat-blast induced production shocks (%)	
			5%	10%
Bangladesh	17.5	-2.84	+1.5%	+2.99%
India	60.6	+2.24	-36.6%	-73.3%
Pakistan	113.6	-0.22	+10.3%	+20.6%

Source: Authors’ calculation, FAO [5].

An increase in wheat imports by Bangladesh and Pakistan, and decreases in India’s exports may increase the price of wheat on the international market. Such increases in price can lead to an increase in the number of people at risk of hunger, not only in South Asia, but also in other countries where wheat is a major staple and they depend on wheat imports. Worryingly, the largest wheat-producing Sub-Saharan African country, Ethiopia, is also vulnerable to the wheat-blast threat due its climatic conditions. In due time, the wheat-blast impacts are likely to reach beyond the already-significant impacts in South Asia.

## Conclusion and policy implications

In 2016, the devastating wheat-blast disease was found in Bangladesh ‒ its first occurrence outside South America. The disease is now endemic in the densely-populated region, presenting potentially devastating negative impacts on wheat production, income and livelihoods of wheat farmers, and the overall food security in Bangladesh.

This study first identified the area vulnerable to wheat blast in South Asia by matching the historical weather variables across Bangladesh, India and Pakistan to those in the 2016 epicenter. The study identified a total of seven million ha of wheat in these countries that are vulnerable to the wheat-blast disease. The study then used two scenarios applied across the vulnerable areas: a 5% and 10% wheat-blast-induced wheat production shock. Across South Asia, the wheat-blast-induced wheat-yield loss scenarios of 5–10% imply a potential wheat loss in affected years of 0.89–1.77 million tons, equivalent to USD 132–264 million. Such losses further threaten the already-precarious national food security, adding pressure to wheat imports and wheat prices. The increase in wheat prices can escalate the number of people facing hunger by lowering their purchasing power, which can ultimately impair the food security of the entire region.

The study is a call for action to tackle the now real wheat-blast threat in South Asia and calls for both short-term and long-term action plans to mitigate the threat. In the short term, investments are needed in research and development to better understand the disease and its implications in the South Asia setting, and to monitor its re-occurrence and resort to immediate control measures to check the spread and damage. Since this is a new disease, awareness creation among wheat growers and all stakeholders is equally important in the short term. Therefore, it is important to develop strategies to manage the disease, such as developing and deploying appropriate fungicides to ensure effective chemicals are available in the hands of the farmers at an affordable price and at the right time, especially during wheat-blast-conducive environments, along with the knowledge of the appropriate use of these fungicides. However, only fungicide application, which is a short-term solution, is not enough as the rapid development of fungicide resistance was observed in Brazil. To prepare for the long term, a concerted effort and long-term assured investments are needed immediately to develop and disseminate blast-resistant wheat varieties, including their targeting to vulnerable areas, and reliable and timely surveillance and disease forecasting to ensure wheat food security for the masses.

The present study has a few limitations. First, data and the associated assumptions intrinsically limit this study. More detailed data sets and enhanced understanding of the disease in the South Asia context could provide the foundation for more rigorous and advanced models in the future (e.g. [40]). This study assumes the potential disease spread in areas based on a similarity of 60% or higher in terms of rainfall and temperature to the 2016 epicenter in Bangladesh. In reality, wheat blast can spread in more restricted or more extended areas than the present homologue areas considered, depending on the local micro-climate conditions which can be more or less favorable to the development of the disease. Using daily climate data for the growing season of the 2016 outbreak sites in Bangladesh would allow the potential high-risk wheat-blast areas to be narrowed down with more precision and allow the establishment of early warning systems to give research institutions, farmers and extension services in the region the ability to react quickly with plant protection measures in wheat fields or spot eradication if needed. Currently, regional coverage of daily data is limited for near real-time warning systems, but investment in the expansion of weather station networks with automated online availability such as is currently being undertaken by the Government of Bangladesh is crucial. Long-term climate conditions were assumed for the analysis, even given the anomaly of the rainfall events leading to the outbreak. However, the reappearance of the pathogen in 2017 shows that, once established, the disease can cause damage even under relatively normal weather conditions similar to the large outbreak in 2009 in South America.

Second, this study assumed some factors to remain constant. For instance, that there will be no gain in virulence in the pathogen, i.e. the disease epidemic in 2016 remains the base. It did not consider any change in acreage of wheat production or its economics due to the presence of the disease and additional costs related to fungicide application and potential price effects. Finally, the origin and pathway of wheat blast into Bangladesh and the pathogen nomenclature are beyond the scope of the present study, although a few studies have linked the Bangladesh introduction with South America [9], probably associated with wheat imports from Brazil. A rigorous establishment of the source and pathways of the wheat-blast disease into Bangladesh and beyond is needed not least to identify the role of quarantine failure and leakages and associated policy learnings and implications to avoid such costly re-occurrences of disease introductions in an ever more connected world.
